# Efficient CRISPR-Mediated Post-Transcriptional Gene Silencing in a Hyperthermophilic Archaeon Using Multiplexed crRNA Expression

**DOI:** 10.1534/g3.116.032482

**Published:** 2016-08-08

**Authors:** Ziga Zebec, Isabelle Anna Zink, Melina Kerou, Christa Schleper

**Affiliations:** Division of Archaea Biology and Ecogenomics, Department of Ecogenomics and Systems Biology, University of Vienna, 1090, Austria

**Keywords:** CRISPR, RNA, gene silencing, hyperthermophile, Archaea, Sulfolobus

## Abstract

CRISPR (Clustered Regularly Interspaced Short Palindromic Repeats)-mediated RNA degradation is catalyzed by a type III system in the hyperthermophilic archaeon *Sulfolobus solfataricus*. Earlier work demonstrated that the system can be engineered to target specifically mRNA of an endogenous host reporter gene, namely the β-galactosidase in *S. solfataricus*. Here, we investigated the effect of single and multiple spacers targeting the mRNA of a second reporter gene, α-amylase, at the same, and at different, locations respectively, using a minimal CRISPR (miniCR) locus supplied on a viral shuttle vector. The use of increasing numbers of spacers reduced mRNA levels at progressively higher levels, with three crRNAs (CRISPR RNAs) leading to ∼ 70–80% reduction, and five spacers resulting in an α-amylase gene knockdown of > 90% measured on both mRNA and protein activity levels. Our results indicate that this technology can be used to increase or modulate gene knockdown for efficient post-transcriptional gene silencing in hyperthermophilic archaea, and potentially also in other organisms.

The discovery of the clustered regularly interspaced short palindromic repeats (CRISPR) and their function in immunity against invading viruses and other genetic elements was certainly one of the major findings in prokaryotic research in recent years (Marraffini 2015). Although it was known that a huge diversity of phages and viruses challenge Bacteria and Archaea in their natural environments (Krupovic *et al.* 2011), the widespread occurrence and the complexity and diversity of these defense systems came as a surprise (Makarova *et al.* 2011). All CRISPR systems share the presence of chromosomal loci encoding spacers homologous to invading DNAs of viruses, plasmids, and other elements, which are separated by iterating repeats (Jansen *et al.* 2002). Upon transcription and processing, the resulting small crRNAs are incorporated into effector protein complexes (Cas complexes) that can be divided into two main classes of different types and subtypes depending on their genomic architecture and their composition (Makarova and Koonin 2015). These complexes are guided through base-pairing of the crRNA to their DNA or RNA targets, with subsequent cleavage of the latter. CRISPR systems of class 2 (type II, putative types V and VI) all target DNA with a single, large, effector protein (Makarova and Koonin 2015; Shmakov *et al.* 2015). Probably the most prominent representative of this class is the effector protein Cas9 (CRISPR type II) (Deltcheva *et al.* 2011; Jinek *et al.* 2012), which is now used widely as a genome editing tool for placing mutations in virtually all eukaryotic model systems, including human cell lines (Cong *et al.* 2013; Mali *et al.* 2013; Shalem *et al.* 2014) and in bacteria (Jiang *et al.* 2013). Cas9 was also elegantly engineered to repress or activate gene transcription in bacteria, and even eukaryotes, through the use of nuclease-deficient Cas9 variants that are guided to genomic sites to physically block regulatory enzymes or the transcription complex itself (Gilbert *et al.* 2013; Qi *et al.* 2013; Konermann *et al.* 2015). Interestingly, type II/Cas9 CRISPR systems have exclusively been found in bacteria, while archaea possess predominantly multiprotein effector complexes categorized into class I CRISPR-Cas systems, such as type I or type III (Makarova and Koonin 2015). While type I complexes (commonly known as CASCADE complexes; CRISPR-associated complex for antiviral defense) exclusively target DNA, type III systems (CSM and CMR) are unique in their ability to also degrade RNA (Makarova and Koonin 2015). A handful of different type III-B (CMR) complexes of hyperthermophilic archaea (*Pyrococcus furiosus* and *Sulfolobus solfataricus*) and bacteria (*Thermus thermophilus* and *Thermotoga maritima*) have been studied *in vitro* and all require crRNAs with 8-nt 5′ repeat handles (derived from the flanking repeat sequence) for targeting RNA complementary to the spacer (Zhang *et al.* 2012; Staals *et al.* 2013; Hale *et al.* 2014; Osawa *et al.* 2015a, 2015b; Taylor *et al.* 2015; Zhang *et al.* 2016, Estrella *et al.* 2016). *In vitro* studies report RNA cleavage of two coexisting type III complexes in *S. solfataricus*. While Sso-IIID (CSM) cleaved both RNA and DNA, Sso-IIIB (CMR) targeted solely RNA (Zhang *et al.* 2012, 2016). Additional to these two type III complexes, *S. solfataricus* carries three type I complexes associated with six CRISPR loci. We have recently established an *in vivo* system for this hyperthermophilic archaeon that enabled the study of not only CRISPR-mediated DNA cleavage (Manica *et al.* 2011, 2013) but also RNA interference (Zebec *et al.* 2014). By allowing for base pairing between the repeat-derived 5′-handle of the crRNA and the protospacer adjacent sequence (PAS) of the targeted virus, we were able to fully suppress DNA interference (Manica *et al.* 2013), as shown first for the bacterium *S. epidermidis* (Marraffini and Sontheimer 2010). Despite inhibiting DNA interference, RNA cleavage was unaffected in *Sulfolobus* by the PAS–handle binding, which was also confirmed *in vitro* for other systems (Tamulaitis *et al.* 2014; Samai *et al.* 2015; Estrella *et al.* 2016). This enabled us to study post-transcriptional RNA-targeting in the organism, and to engineer the system for silencing a chromosomal gene, β-galactosidase (Zebec *et al.* 2014). Using an analogous strategy, Peng and coworkers have achieved *in vivo* RNA targeting in the related strain *S. islandicus*, which contains two type III-B complexes, one of which additionally degrades DNA in a transcription-dependent manner (Deng *et al.* 2013; Peng *et al.* 2015).

These observations demonstrate that efficient post-transcriptional RNA-targeting technology can, in principle, be developed from type III systems of hyperthermophilic organisms. In our initial studies, we introduced an artificial miniCRISPR locus that contained a single cognate spacer for the endogenous β-galactosidase gene, resulting in a 50% reduction of the targeted mRNA and the corresponding intracellular protein activity (Zebec *et al.* 2014). Here, we demonstrate that the system can be applied to post-transcriptional RNA targeting of another endogenous chromosomal gene (α-amylase), and that mRNA degradation can be increased gradually using miniCRISPRs with multiple spacers against a single targeted mRNA, achieving an up to > 90% gene knockdown, as reflected by mRNA levels and protein activity.

## Materials and Methods

### Culturing and transfection

The uracil-auxotrophic *S. solfataricus* P1 mutant strain M18 (Martusewitsch *et al.* 2000) was grown at 78° and pH 3 in basic Brock media (Brock *et al.* 1972) supplemented with 0.1% tryptone (Roth) and 0.2% (+) d-sucrose (Serva) (w/v). Untransformed cells were supplied with uracil (Sigma-Aldrich) at a final concentration of 0.0125 mg/ml to complement for uracil auxotrophy. M18 cells were transformed with the pDEST-MJ-miniCR virus shuttle vector variants via electroporation as described earlier (Manica *et al.* 2011). An inverse plaque assay was performed after electroporation in which transformants were mixed with M18 cells in a 0.4% Brock-media gellan gum (GELRITE, Roth) solution, and poured on uracil-free plates. Different to previous plaque assays with wild-type cells in the overlay (Manica *et al.* 2013), auxotrophic cells were used, guaranteeing growth and subsequent plaque formation of only infected cells. Three plaques per transformation were isolated and transferred separately (as biological triplicates) into fresh Brock/tryptone medium to which starch (Roth, starch soluble) was added at a final concentration of 0.04% (w/v). These cultures were grown until stationary phase, and each replicate was transferred into fresh medium. Cell growth was monitored carefully, and samples were taken at regular intervals for DNA and RNA extractions as well as for protein assays (see below).

### Nucleic acid extraction and cDNA preparation

Five milliliters of liquid cell cultures of *S. solfataricus* M18 transformants sampled at early (t1: OD_600_ = 0.1–0.15) and late (t2: OD_600_ = 0.4–0.45) logarithmic growth were used for DNA and RNA extraction respectively, as described in (Zebec *et al.* 2014). Isolated DNA was treated with RNase (Omega, bio-tek) before further analysis. RNA was further treated with RQ1-DNAse I (Promega) to digest potential traces of DNA, and purity was verified by a polymerase chain reaction (PCR). Nucleic acid concentrations were determined by NanoDrop (ND-1000, PeqLab) measurements. cDNA was reverse transcribed from 1 µg of RNA using the ProtosScript II Reverse Transcriptase (New England BioLabs) following the manufacturer’s instructions, and then column-purified (NucleoSpin, Macherey-Nagel); 20 ng/µl dilutions of cDNA were used in real-time PCR quantification (qPCR).

### Construction of miniCR vectors

Construct pENTRY NBG (Zebec *et al.* 2014) carrying a 900 bp region of CRISPR locus D of *S. solfataricus* P2 (consisting of a 497 bp part of the locus D leader and the first six spacers, interspaced by locus D-repeats) served as a template vector for the construction of the multiple spacer miniCR variants. A modulated version of an overlap extension PCR (OE PCR) (Bryksin and Matsumura 2010) was used to synthesize miniCR spacer constructs with up to five spacers (AA1, AA2, AA3, AA13, AA23, AA123, and AA12345), and the control vector pZ2, respectively (Supplemental Material File S1 and, Figure S2). For fusion of primers and amplification in all the following PCR reactions, a proofreading polymerase was used (High fidelity Phusion DNA polymerase, Thermo Scientific). With this cloning strategy, spacers D2, D3, and D4 were replaced as described in (Zebec *et al.* 2014) by PCR fusion of a 50 nt long MOE primer (MOE-Fw matching spacer D1, and MOE-Rw matching spacer D5), and specific AA primers designed to match the selected α-amylase protospacers (Figure S2). In the first PCR step, two primers annealed to the repeat sequence (called flank), and were extended by the DNA polymerase, starting from the middle of the flank region. Flanks also contained a specific sequence, the spacer, on each side, that represented the unique part of the flank (Figure S2A) programmed to match the selected α-amylase protospacers (spacer selection was based on the presence of PAS). Each synthesized flank was then purified using a PCR clean-up column (NucleoSpin, Macherey-Nagel). In step 2 PCR, this unique part of the flank allowed us to specifically fuse together any number of flanks to form an OE fragment. The step 2 PCR not only fused the flanks into a longer OE fragment, it also amplified the OE fragment with the primer pair (called M), which binds to the specific spacer part of the MOE primers. In this manner, the OE fragment was amplified only if flanks were correctly fused. The third and last sequential PCR used the OE fragment as a primer (ratio primer: pEntry = 200:1, with 20–30 ng OE fragment, and 80–100 ng of any pEntry vector) on the pEntry vector containing a miniCR region, as described for miniCRISPR-BG-HA (Zebec *et al.* 2014). After the OE fragments annealed to the vector in the MOE section (spacer D1 and D5), all new artificial spacers were inserted into the new construct between spacer D1 and D5 (between MOE-Fw and MOE-Rw). The control construct pZ2 (Figure S1) was assembled in the same manner, with the sole difference that the nonsense spacers Z1 and Z2 (each 39 nt) did not match the α-amylase transcripts (> 20 mismatches at any place on the target gene). Following step 3 PCR, the product was directly digested with *Dpn*I restriction enzyme (New England BioLabs) in order to eliminate false positive clones that contained the *dam* methylated template pEntry vector originating from *Escherichia coli* (TOP10). This procedure was highly specific, and yielded only correctly amplified PCR constructs.

Constructing the miniCR-MA2 harboring three identical spacers (AA2) was more challenging, since the two inner flanks (which are not fused to MOE primers) did not contain a specific part representing a unique spacer. To overcome this problem, a circular miniCR-AA2 was first constructed that contained only one AA2 spacer (as described above). In the next step, the plasmid miniCR-AA2 was linearized in the middle of the spacerAA2 by inverse PCR (step 1), using the primers MA2_lin (Table S1). The linear miniCR-AA2 contained half of the AA2 spacer on each side of the plasmid, and was used as template in the successive PCR reaction (step 2). The primers used in step 2 PCR were 80 nt long MA2_over primers (corresponding to one-and-a-half AA2 spacer), which annealed to the AA2 side of the linear template. In order to minimize self-polymerization of the MA2_over primers and chimera production with the template, the second PCR step included only four cycles of PCR amplification followed by a PCR clean-up procedure (NucleoSpin, MACHEREY-NAGEL). The final circularization of the miniCR-MA2 was carried out with Quick-Ligase (New England BioLabs). The circular, *E. coli*-originating miniCR-AA2 template plasmid was digested with *Dpn*I (New England BioLabs) as described above. Through this procedure, one in four *E. coli* clones tested harbored the correct construct. Plasmids were recovered and miniCR regions were inserted into the final virus shuttle-vector pDEST-MJ (Zebec *et al.* 2014) of *S. solfataricus* via Gateway *in vitro* recombination (Invitrogen, Life technologies).

### Quantitative PCR analysis

DNA gene copies and reverse transcribed mRNA (cDNA) quantification was performed with qPCR on an Eppendorf Mastercycler ep*gradient* S realplex^2^ (Eppendorf). Three biological replicates and three technical replicates were measured, respectively, for each sample and control presented in this study. The standard used in each qPCR run was the enzymatically (*Xho*I, Thermo Scientific) linearized, 22 kb viral vector pDEST-MJ (Zebec *et al.* 2014), which contained the α-amylase gene. To quantify the DNA copies of the viral open reading frame A291, the primer pair Q-A291 was used, and α-amylase (SSO1172) gene copies were quantified with the primer pair QAA2-sp (Table S1 and Table S2). The housekeeping gene used as a reference of general transcription and cDNA synthesis efficiency was SSO3194 (encoding glyceraldehyde-3-phosphate dehydrogenase). Primer pairs target specifically QAA2-sp and the reference gene QSSO3194 (Table S1 and Table S2), respectively, and were used to measure transcripts from the same cDNA preparation, for each sample and control. For each construct, ΔC_t_ of the two primer pairs (SSO3194 and QAA2-sp) was calculated and then normalized to the values of the control construct pZ2, which contained two nonsense spacers (significance values in Figure 3). The qPCR efficiency was between 94% and 100%, in all qPCR runs. Primer sequences and amplicon lengths are listed in Table S1.

### Protein/α-amylase activity assay

Hydrolytic activity of the secretory protein α-amylase (SSO1172 or SSO_RS05765) on starch was determined by the photometric quantification of iodine binding to starch, as described by Haseltine *et al.* (1996) and Xiao *et al.* (2006), with the following modifications. Culture supernatant samples were taken at regular intervals during the course of the growth curve, between OD 0.05 and 0.4, and stored at 4° until processing. Quantification of the remaining starch in the cultures was performed by adjusting 162 μl of sample to pH 3.5 with 10 mM sodium acetate (pH 3.5), and addition of 10 μl of a 1:10 dilution of Lugol solution (Sigma) for color development. Sample absorbance was determined at a wavelength of 580 nm, and was corrected for medium absorbance (deducing the absorbance measured in the media without cells). Consumption was calculated in picograms starch hydrolyzed per chromosome (as calculated by qPCR) in the course of the growth curve [*i.e.*, measuring consumption between t0 (OD = 0.05) and t2 (OD = 0.45)], and is represented relative to the control culture pZ2. At least three biological and three technical replicates were measured for each miniCR construct, respectively.

The provided Supplemental Material includes Figure S1, Figure S2A, Figure S2B, Figure S3, Figure S4, Table S1, Table S2, and File S1. Figure S1 contains a schematic representation of the multiplex miniCR constructs analyzed in this study. Figure S2 contains a schematic overview of the modular OE-PCR. Figure S3 contains the sequence of miniCR-AA12345. Figure S4 illustrates the quantification of viral copies per chromosome. Table S1 and Figure S2 contain information on the PCR reactions and primers used in this study.

### Data availability

The authors state that all data necessary for confirming the conclusions presented in the article are represented fully within the article.

## Results

**Figure 1:**
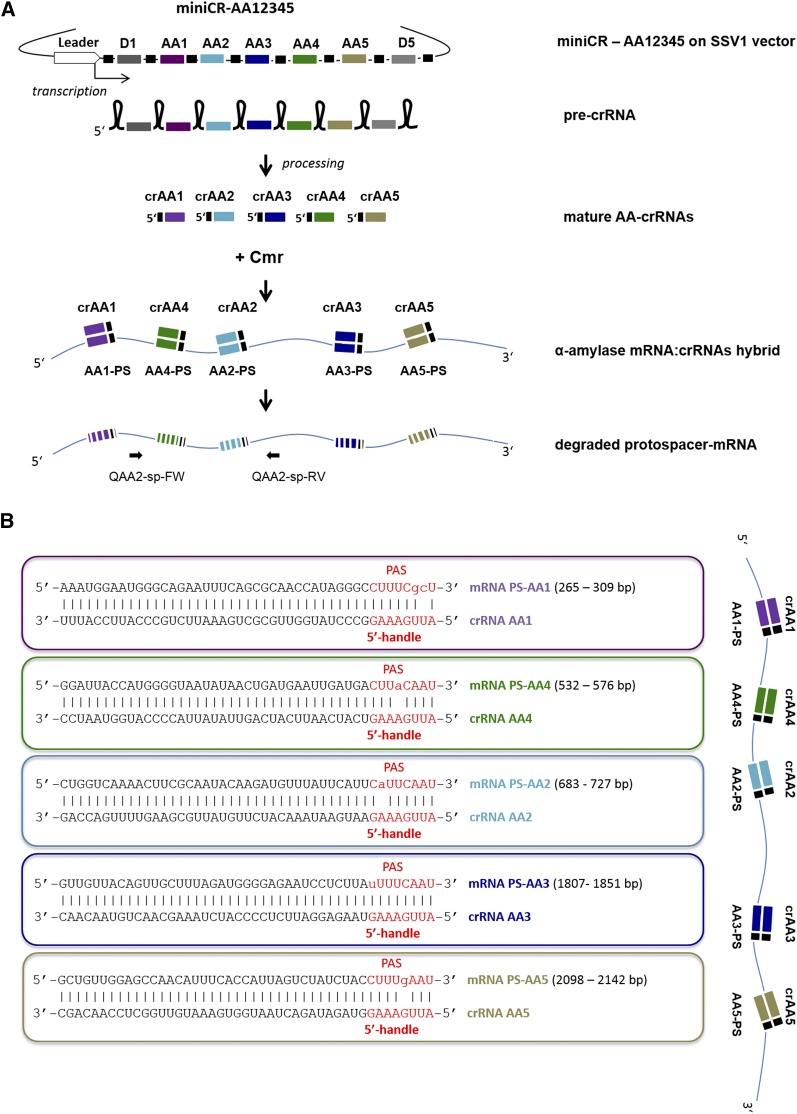
(A) Schematic representation of programmed silencing of the α-amylase of *S. solfataricus* in a transfection assay using a multiplex miniCRISPR locus (miniCR-AA12345). Locations of primers used in qPCR are indicated (Q-AA2-sp-FW and Q-AA2-sp-RV). (B) Protospacer (*i.e.*, targeting sites) regions in α-amylase mRNA with 3′ PAS hybridizing to matching crRNA and 5′ handle. Exact protospacer positions on mRNA sequence are indicated. Upper case letters, complementarity; lower case letters, mismatch; linked lower case letters, G:U pairing through mismatch.

In our previous work, we were able to knock down the β-galactosidase gene expression of *S. solfataricus* to 50% (measured on mRNA and protein level) by expressing an engineered miniCR locus carrying a single spacer against the chromosomally encoded β-galactosidase mRNA (Zebec *et al.* 2014). This miniCR was derived from CRISPR locus D of *S. solfataricus*, and contained additionally five original flanking spacers and the leader sequence (encoding a transcriptional promoter). Using an analogous strategy, we focused here on the modulation of the miniCR system determining the silencing efficiency of another host-derived gene using multiple targeting spacers expressed from a single miniCR construct. As a target, the α-amylase of *S. solfataricus* was selected—a secreted enzyme catalyzing the hydrolysis of polymeric starch to linear maltodextrins (Worthington *et al.* 2003). The secreted protein was identified by mass spectrometry from an SDS-PAGE of culture supernatant as the verified gene product of the 2709 bp gene SSO1172 (or SSO_RS05765) encoding α-amylase (not shown). Five 37-bp sequences were chosen as protospacers (*i.e.*, target sequences) on the α-amylase mRNA, according to which the miniCR spacers AA1–AA5 were designed (Figure 1, A and B). The protospacers were chosen such that their adjacent 3′ sequences (PAS) matched at least 6 nt of the 5′-handle of the crRNAs (which is derived from the repeat sequences of the CRISPR locus, and is used for chromosome (“self”) recognition and protection (Figure 1B). We have previously shown that matches of only 3 bp between the PAS and the 5′ handle at distinct positions (–3, –4, –5) were sufficient to completely suppress CRISPR-mediated DNA cleavage (Manica *et al.* 2013). A systematic survey of the *S. solfataricus* chromosome revealed that this 3-nt long PAS motif is found at least once in 99% of all CDS (coding DNA sequence), whereof 85% contain at least five occurrences of the PAS motif. This indicates that cognate crRNAs for gene silencing, while circumventing DNA degradation, can be easily designed for every gene in the host chromosome. The following miniCR constructs, each carrying a different number and composition of spacers were designed: three single constructs carrying one spacer each (miniCR-AA1, -AA2, -AA3), two double constructs harboring two spacers each (miniCR-AA13 and miniCR-AA23), miniCR-AA123 carrying three different spacers, and miniCR-AA12345 carrying five spacers targeting α-amylase at five different positions (Figure 1B and Figure S1). In addition, construct miniCR-MA2 carried spacer AA2 three times in series (interspaced by repeats), aiming to analyze the impact of the dosage of a single spacer on the silencing effect (Figure 1B and Figure S1). As a control, construct pZ2, which carries a miniCR backbone with two spacers (Z1 and Z2) not matching α-amylase, was designed. All miniCR constructs were inserted into the SSV1-virus shuttle vector pDEST-MJ (Zebec *et al.* 2014), and the resulting recombinant shuttle virus was used to transfect *S. solfataricus*. Transfected cells were used in an inverted plaque assay (*Materials and Methods*), and one plaque of each transfectant was transferred into Brock growth medium supplied with starch to induce expression of α-amylase (Grogan 1989).

The SSV1 virus-based shuttle vector has been used repeatedly for genetic manipulation of *S. solfataricus* (Schleper *et al.* 1992, Jonuscheit *et al.* 2003, Albers *et al.* 2006, Manica *et al.* 2011, 2013), because propagation of the construct is more efficient compared to a cryptic plasmid-based system, but the viral DNA nevertheless remains in stable copy numbers. To verify that each miniCR-transformant carried a similar amount of viral DNA copies, *i.e.*, same amount of miniCRs, virus copies per cell (per host chromosome) had to be determined. For this, total DNA was isolated from each culture at t1 and t2 (early- and late-exponential growth phase), respectively, and analyzed by qPCR using primers specific for the host chromosome (AA-Q2-no sp, Table S1) and the viral genome (Q-A291, Table S1), respectively, on the same DNA sample. The average viral counts per chromosome were constant between cultures, at 1–2 copies per chromosome (six replicates of each construct were tested, Figure S4).

**Figure 2:**
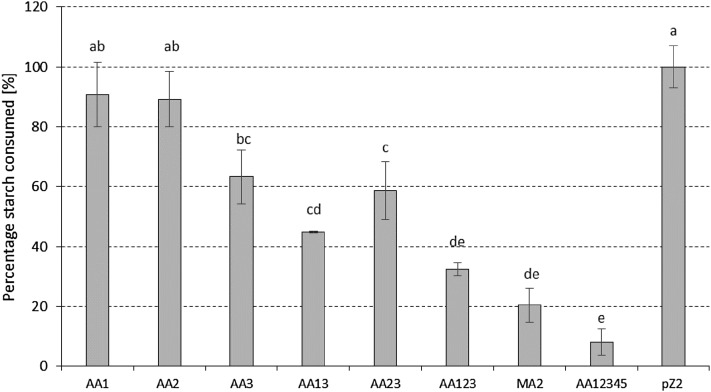
Starch consumption expressed as percentage of the control (pZ2) measured over the course of growth in cultures of transformants, as described in *Materials and Methods*. At least three biological replicates with three technical replicates were measured in each case. Error bars show SE (*n* ≥ 3). Lowercase letters indicate significant differences between sample means (Fisher’s least significant difference, LSD, *P* ≤ 0.05), *i.e.*, same letters show no significant difference of means. AA1/AA2/AA3, miniCR carrying one spacer each; AA13/AA23, miniCR carrying two different spacers; AA123, miniCR carrying three different spacers; MA2, miniCR carrying spacer AA2 three times; AA12345, miniCR carrying five different spacers.

Gene silencing by the different miniCR constructs was determined at the protein level, where the remaining starch content in the medium was quantified (over growth from OD_600_ = 0.05 until late exponential phase, OD_600_ = 0.45) as a measure of the hydrolytic activity of the secreted α-amylase protein. The measurements were compared to the control pZ2, where no decrease in starch consumption (*i.e.*, no silencing) was observed (Figure 2). Our results demonstrate the trend of a gradual increase of silencing with an increasing number of spacers targeting the α-amylase at different positions. Single spacer constructs showed the weakest silencing effect of around 10% for AA1 and AA2, respectively, and around 35% for AA3, when compared to pZ2. An increase in silencing activity was observed for double constructs AA13 and AA23, which exhibited a decrease of 55% and 41% in starch consumption, respectively. Hydrolytic activity of α-amylase was further reduced for construct AA123, carrying spacers AA1, AA2, and AA3, to around 30% residual activity. For MA2, the triple-dosage construct, carrying the spacer AA2 three times, 80% silencing was determined compared to the activity of the pZ2 control. Consumption was drastically repressed with construct AA12345, where cells consumed overall only around 10% (*i.e.*, 90% silencing) of starch compared to the control (Figure 2), *i.e.*, 90% silencing effect.

**Figure 3:**
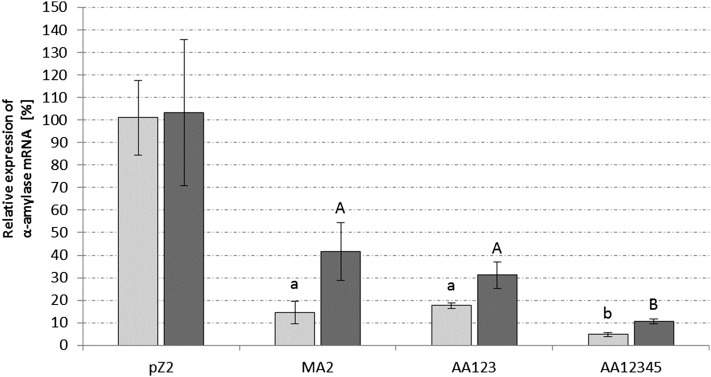
Relative quantification of α-amylase mRNA via qPCR. Light gray columns represent samples taken in the early exponential growth stage, and dark gray represents samples taken in the late exponential growth stage. The primer pair QAA2-sp was used to measure α-amylase mRNA levels amplifying the region of AA2 and AA4 (Figure 1, A and B). The gene SSO3194, coding for glyceraldehyde-3-phosphate dehydrogenase, was used as a housekeeping gene. Three biological replicates were measured with three technical replicates, for each construct, respectively. All values present relative expression of α-amylase mRNA compared to control miniCR-pZ2 in percent. Significance to the control pZ2 was determined by a one-tailed *t*-test, *n* ≥ 3, with *P* ≤ 0.0063 for early growth stage (lowercase letters) and *P* ≤ 0.032 for the late growth stage (capital letters). Error bars represent SD (*n* ≥ 3). No significant difference between early and late growth stage was observed for the control construct pZ2.

To verify whether the silencing effect observed in the protein assay was reflected in the mRNA level, α-amylase transcripts were quantified via qPCR. RNA was sampled, reverse transcribed, and analyzed from early exponential growth phase (t1, OD_600_ = 0.15) and late growth phase (t2, OD_600_ = 0.45) of cultures showing the strongest silencing effect on the protein level (miniCR-AA123, miniCR-MA2, and miniCR-AA12345). The primer pair Q-AA2-sp was used for quantification of α-amylase in relation to transcripts of the chromosomally encoded SSO3194 gene used as reference (Pfaffl 2001). In samples taken during the early growth stage, we detected a relative decrease of 85% in α-amylase mRNA for the dosage construct miniCR-MA2, and 82% lower mRNA levels were measured for construct AA123. A 95% decrease in α-amylase mRNA molecules were detected for AA12345 (Figure 3). During late exponential phase, the decrease in α-amylase mRNA compared to the control pZ2 was generally lower, but still significant. Overall, highest RNA degradation of 95% in the early growth stage, and 90% in the late growth stage, was achieved with construct AA12345. These findings were in agreement with the reduced protein levels in the same transformants (Figure 2).

## Discussion

In this study, we were able to silence the α-amylase gene of *S. solfataricus* up to 95% using a minimal CRISPR version transcribed from a native leader carrying artificial spacers against the amylase mRNA (Figure 2 and Figure 3). A gradual increase of the silencing effect from maximally 35% with a single spacer, to 95% with a quintuple spacer arrangement, was observed.

Three different single-spacer miniCR constructs were compared in this study. Surprisingly, two of them (AA1 and AA2), showed no significant silencing effect compared to control cultures, whereas AA3 showed around 35% silencing (Figure 2). These results differ from our previous observations, where only one spacer expressed from the same miniCR backbone was sufficient to knock down the β-galactosidase in *S. solfataricus* to 50% (Zebec *et al.* 2014). Since silencing requires both the successful incorporation of crRNA into a type III silencing complex, and the capability of crRNA to bind efficiently to its target site, we assume that one or both of these mechanisms was less efficient for AA1 and AA2. It seems to be difficult to predict the position on the mRNA (*i.e.*, protospacer) that leads to most efficient silencing when targeted, since internal structures of RNAs and hybridization parameters are challenging to determine accurately. However, by calculating interaction energies between the single crRNAs and their protospacers on the mRNA, we found them to be thermodynamically favorable (dG < –22 kcal/mol) over any RNA secondary structures inhibiting binding (calculated with RNAup—ViennaRNAPackage Version 2.1.9; Mückstein *et al.* 2006). Further experiments are needed to systematically address the parameters determining the effectiveness of a given spacer in order to improve spacer design. Nevertheless, when combining the AA1 and AA2 spacers with AA3 to produce double (AA13 and AA23) or triple (AA123) constructs, the silencing effect increased up to 70% (Figure 2). Interestingly, an excess of spacer AA2 expressed via construct MA2 (carrying the AA2 spacer three times) also proved to be extremely efficient, resulting in 80% reduction in α-amylase activity. The dosage impact of crRNAs was also observed in the study of Peng *et al.* (2015), in which the overexpression of one spacer by a strong promoter resulted in 90% silencing of a reporter gene in *S. islandicus*.

During the early growth stage there seemed to be no difference in CRISPR-mediated RNAi (RNA interference) strength when three spacers were used as in constructs AA123 and MA2 (Figure 3). Around 85% of α-amylase mRNA was reduced regardless of whether the spacers were targeting multiple positions (AA123) or a single one (MA2). This effect is also retained in the late exponential growth stage, although overall silencing was weaker (around 60–70%) which is probably due to a general loss of cell fitness, or the accumulated effect of the highly stable active enzyme produced by transcripts that escaped silencing (Figure 3). Overall, it seems that there is no obvious difference in silencing strength between multiple site or single site targeting.

Multiple site targeting (such as from miniCR-AA123, or miniCR-AA12345) might be advantageous for targeted gene silencing, since hybridization of one crRNA to its binding site might promote the accessibility of a second binding site elsewhere on the mRNA though conformational changes. Furthermore, it might also reflect the natural targeting strategy of the CRISPR in environmental systems, since *de novo* spacers seem to be acquired from different loci of invading genomes than repeatedly from the same site, as illustrated by the uptake of multiple spacers matching a single genetic element within CRISPR arrays in *Sulfolobales* (Held *et al.* 2010; Erdmann *et al.* 2013; Erdmann and Garrett 2015; Levy *et al.* 2015). Nevertheless, in our experiment, miniCR-MA2 showed that dosage can have a strong effect, even if the effectiveness of the respective single spacer alone is relatively low. Therefore, an overexpression of single crRNAs might be interesting if small RNA molecules that do not contain more than one minimal PAS are targeted and, therefore, cannot be tackled in multiple positions.

The strong RNAi effect of the quintuple construct AA12345 was similar in early and late growth stages. These data suggest that five targets in a single RNA molecule (or less, depending on the choice of crRNAs) are sufficient to effectively degrade a specific RNA. In order to further improve this system for silencing of essential genes, it will be useful to employ inducible promoters for expression of the engineered miniCRs. It will also be important to investigate the extent of cross-reactions of the crRNAs with other targets, *i.e.*, to determine the extent of off-targeting.

From a mechanistic perspective, it would be important and interesting to reveal the cross-talk and interplay between the five different CRISPR systems in *S. solfataricus*. Even though we can rule out any CRISPR–DNA interference activity by type I and, presumably, type III-D systems using PAS-handle complementarity, we cannot clearly distinguish between the coexisting type III complexes (III-D and III-B) with respect to RNA cleavage. *In vitro* studies show that, in principle, both complexes of *S. solfataricus* cleave RNA, but, so far, it has only been shown for the CMR III-B system that crRNAs expressed from our miniCRISPR are incorporated and used as a guide to cleave the target (Zhang *et al.* 2016; Zebec *et al.* 2014). Due to the lack of efficient knockout techniques in this strain, we could not construct CRISPR mutants or nuclease deficient complexes in *S. solfataricus* to address this question *in vivo*.

Overall, we have demonstrated a gene silencing effect of up to 90–95% on the mRNA level, and 90% on the protein level while using five spacers (AA12345) against the α-amylase mRNA molecules. We have shown that, besides β-galactosidase, potentially any gene can be efficiently silenced in this host using multiplexed miniCR systems, by appropriating the number of spacers used to the length of the gene. In addition, we have exploited the inherent properties of the CRISPR system to modulate transcript levels by using an increasing number of spacers. This revealed that weak effects of single spacers can be enhanced gradually, either by increasing the total yield of crRNA, or through combinations of different spacers.

### Conclusions

Besides groundbreaking genome-editing tools derived from CRISPR types II and V (Jinek *et al.* 2012; Fagerlund *et al.* 2015; Zetsche *et al.* 2015; Wright *et al.* 2016), more progress is now being made in modulating and exploiting type I and type III CRISPR complexes for genetic manipulation (Rath *et al.* 2015; Luo *et al.* 2015) . Only recently, Li *et al.* (2015) elegantly used the DNA targeting complexes CASCADE and CMR-α complexes to set mutations in genes of interest in the *S. islandicus* genome.

Recent research has also presented the huge potential for other CRISPR systems to alter cell or viral mRNA levels, *e.g.*, the recently described Class 2 type VI-A system C2c2 (Abudayyeh *et al.* 2016). The dCas9 system is a leading, and readily commercially available, silencing and activation system that can be applied in most organisms (bacteria and eukarya), except extremophiles, to which most cultivated archaea belong (Qi *et al.* 2013; Gilbert *et al.* 2014; Konermann *et al.* 2015). No dCas9 system was reported or introduced into Archaea, which also lack the RNase III activity needed for crRNA processing in Cas9 systems. Considering the results of this study and that of Peng *et al.* (2015), it now seems possible to conduct efficient gene silencing by targeting transcripts of any size in *Sulfolobus*, and to any desired level for investigating phenotypical effects. The miniCR system (Zebec *et al.* 2014; Peng *et al.* 2015), combined with a type III activity, represents the first tool that allows post-transcriptional gene silencing by directly attacking RNA independently of DNA binding, and thus differs from the type II, dCas9 CRISPR-systems. Furthermore, it represents the first gene silencing mechanism that can be used in hyperthermophilic microorganisms, for which genetic tools are generally scarce. Being able to silence genes in *Sulfolobus* means it becomes possible to study the function of essential genes involved in central information processing in archaea, which are highly conserved between archaea and eukaryotes.

## 

## Supplementary Material

Supplemental Material

## References

[bib1] Abudayyeh O. O. Gootenberg J. S. Konermann S. Joung J. Slaymaker I. M., 2016 C2c2 is a single-component programmable RNA-guided RNA-targeting CRISPR effector. Science 353: aaf5573. 10.1126/science.aaf5573 PMC5127784 27256883

[bib2] Albers S. V. Jonuscheit M. Dinkelaker S. Urich T. Kletzin A., 2006 Production of recombinant and tagged proteins in the hyperthermophilic archaeon *Sulfolobus solfataricus*. Appl. Environ. Microbiol. 72(1): 102–111. 16391031 10.1128/AEM.72.1.102-111.2006 PMC1352248

[bib3] Brock T. D. Brock K. M. Belly R. T. Weiss R. L., 1972 *Sulfolobus*: a new genus of sulfur-oxidizing bacteria living at low pH and high temperature. Arch. Mikrobiol. 84: 54–68. 4559703 10.1007/BF00408082

[bib4] Bryksin A. V. Matsumura I., 2010 Overlap extension PCR cloning: a simple and reliable way to create recombinant plasmids. Biotechniques 48: 463–465. 20569222 10.2144/000113418 PMC3121328

[bib5] Cong L. Ran F. A. Cox D. Lin S. Barretto R., 2013 Multiplex genome engineering using CRISPR/Cas systems. Science 339: 819–823. 23287718 10.1126/science.1231143 PMC3795411

[bib6] Deltcheva E. Chylinski K. Sharma C. M. Gonzales K. Chao Y., 2011 CRISPR RNA maturation by trans-encoded small RNA and host factor RNase III. Nature 471: 602–607. 21455174 10.1038/nature09886 PMC3070239

[bib7] Deng L. Garrett R. A. Shah S. A. Peng X. She Q., 2013 A novel interference mechanism by a type IIIB CRISPR-Cmr module in *Sulfolobus*. Mol. Microbiol. 87: 1088–1099. 23320564 10.1111/mmi.12152

[bib8] Erdmann S. Garrett R. A., 2015 Archaeal viruses of the Sulfolobales: isolation, infection, and CRISPR spacer acquisition. Methods Mol. Biol. 1311: 223–232. 25981476 10.1007/978-1-4939-2687-9_14

[bib9] Erdmann S. Shah S. A. Garrett R. A., 2013 SMV1 virus-induced CRISPR spacer acquisition from the conjugative plasmid pMGB1 in *Sulfolobus solfataricus* P2. Biochem. Soc. Trans. 41: 1449–1458. 24256236 10.1042/BST20130196 PMC3839810

[bib10] Estrella M. A. Kuo F. T. Bailey S., 2016 RNA-activated DNA cleavage by the Type III-B CRISPR-Cas effector complex. Genes Dev. 30: 460–470. 26848046 10.1101/gad.273722.115 PMC4762430

[bib11] Fagerlund R. D. Staals R. H. Fineran P. C., 2015 The Cpf1 CRISPR-Cas protein expands genome-editing tools. Genome Biol. 16: 251. 26578176 10.1186/s13059-015-0824-9 PMC4647450

[bib12] Gilbert L. A. Larson M. H. Morsut L. Liu Z. Brar G. A., 2013 CRISPR-mediated modular RNA-guided regulation of transcription in eukaryotes. Cell 154: 442–451. 23849981 10.1016/j.cell.2013.06.044 PMC3770145

[bib13] Gilbert L. A. Horlbeck M. A. Adamson B. Villalta J. E. Chen Y., 2014 Genome-scale CRISPR-mediated control of gene repression and activation. Cell 159: 647–661. 10.1016/j.cell.2014.09.029 PMC4253859 25307932

[bib14] Grogan D. W., 1989 Phenotypic characterization of the archaebacterial genus *Sulfolobus*: comparison of five wild-type strains. J. Bacteriol. 171: 6710–6719. 2512283 10.1128/jb.171.12.6710-6719.1989 PMC210567

[bib15] Hale C. R. Cocozaki A. Li H. Terns R. M. Terns M. P., 2014 Target RNA capture and cleavage by the Cmr type III-B CRISPR-Cas effector complex. Genes Dev. 28: 2432–2443. 25367038 10.1101/gad.250712.114 PMC4215187

[bib16] Haseltine C. Rolfsmeier M. Blum P., 1996 The glucose effect and regulation of alpha-amylase synthesis in the hyperthermophilic archaeon *Sulfolobus solfataricus*. J. Bacteriol. 178: 945–950. 8576067 10.1128/jb.178.4.945-950.1996 PMC177752

[bib17] Held N. L. Herrera A. Cadillo-Quiroz H. Whitaker R. J., 2010 CRISPR associated diversity within a population of *Sulfolobus islandicus*. PLoS One 5: e12988. 10.1371/journal.pone.0012988 PMC2946923 20927396

[bib18] Jansen R. Embden J. D. Gaastra W. Schouls L. M., 2002 Identification of genes that are associated with DNA repeats in prokaryotes. Mol. Microbiol. 43: 1565–1575. 11952905 10.1046/j.1365-2958.2002.02839.x

[bib19] Jiang W. Bikard D. Cox D. Zhang F. Marraffini L. A., 2013 RNA-guided editing of bacterial genomes using CRISPR-Cas systems. Nat. Biotechnol. 31: 233–239. 23360965 10.1038/nbt.2508 PMC3748948

[bib20] Jinek M. Chylinski K. Fonfara I. Hauer M. Doudna J. A., 2012 A programmable dual-RNA-guided DNA endonuclease in adaptive bacterial immunity. Science 337: 816–821. 22745249 10.1126/science.1225829 PMC6286148

[bib21] Jonuscheit M. Martusewitsch E. Stedman K. M. Schleper C., 2003 A reporter gene system for the hyperthermophilic archaeon *Sulfolobus solfataricus* based on a selectable and integrative shuttle vector. Mol. Microbiol. 48(5): 1241–1252. 12787352 10.1046/j.1365-2958.2003.03509.x

[bib22] Konermann S. Brigham M. D. Trevino A. E. Joung J. Abudayyeh O. O., 2015 Genome-scale transcriptional activation by an engineered CRISPR-Cas9 complex. Nature 517: 583–588. 25494202 10.1038/nature14136 PMC4420636

[bib23] Krupovic M. Prangishvili D. Hendrix R. W. Bamford D. H., 2011 Genomics of bacterial and archaeal viruses: dynamics within the prokaryotic virosphere. Microbiol. Mol. Biol. Rev. 75: 610–635. 22126996 10.1128/MMBR.00011-11 PMC3232739

[bib24] Levy A. Goren M. G. Yosef I. Auster O. Manor M., 2015 CRISPR adaptation biases explain preference for acquisition of foreign DNA. Nature 520: 505–510. 25874675 10.1038/nature14302 PMC4561520

[bib25] Li Y. Pan S. Zhang Y. Ren M. Feng M., 2015 Harnessing Type I and Type III CRISPR-Cas systems for genome editing. Nucleic Acids Res. 44: e34. 10.1093/nar/gkv1044 PMC4770200 26467477

[bib26] Luo M. L. Mullis A. S. Leenay R. T. Beisel C. L., 2015 Repurposing endogenous type I CRISPR-Cas systems for programmable gene repression. Nucleic Acids Res. 43: 674–681. 25326321 10.1093/nar/gku971 PMC4288209

[bib27] Makarova K. S. Koonin E. V., 2015 Annotation and classification of CRISPR-Cas systems. Methods Mol. Biol. 1311: 47–75. 25981466 10.1007/978-1-4939-2687-9_4 PMC5901762

[bib28] Makarova K. S. Haft D. H. Barrangou R. Brouns S. J. Charpentier E., 2011 Evolution and classification of the CRISPR-Cas systems. Nat. Rev. Microbiol. 9: 467–477. 21552286 10.1038/nrmicro2577 PMC3380444

[bib29] Mali P. Yang L. Esvelt K. M. Aach J. Guell M., 2013 RNA-guided human genome engineering via Cas9. Science 339: 823–826. 23287722 10.1126/science.1232033 PMC3712628

[bib30] Manica A. Zebec Z. Teichmann D. Schleper C., 2011 In vivo activity of CRISPR-mediated virus defence in a hyperthermophilic archaeon. Mol. Microbiol. 80: 481–491. 21385233 10.1111/j.1365-2958.2011.07586.x

[bib31] Manica A. Zebec Z. Steinkellner J. Schleper C., 2013 Unexpectedly broad target recognition of the CRISPR-mediated virus defence system in the archaeon *Sulfolobus solfataricus*. Nucleic Acids Res. 41: 10509–10517. 24021627 10.1093/nar/gkt767 PMC3905844

[bib32] Marraffini L. A., 2015 CRISPR-Cas immunity in prokaryotes. Nature 526: 55–61. 26432244 10.1038/nature15386

[bib33] Marraffini L. A. Sontheimer E. J., 2010 Self *vs.* non-self discrimination during CRISPR RNA-directed immunity. Nature 463: 568–571. 20072129 10.1038/nature08703 PMC2813891

[bib34] Martusewitsch E. Sensen C. W. Schleper C., 2000 High spontaneous mutation rate in the hyperthermophilic archaeon *Sulfolobus solfataricus* is mediated by transposable elements. J. Bacteriol. 182: 2574–2581. 10762261 10.1128/jb.182.9.2574-2581.2000 PMC111323

[bib35] Mückstein U. Tafer H. Hackermüller J. Bernhart S. H. Stadler P. F., 2006 Thermodynamics of RNA-RNA binding. Bioinformatics 22: 1177–1182. 16446276 10.1093/bioinformatics/btl024

[bib36] Osawa, T., H. Inanaga, and T. Numata, 2015a Crystallization and preliminary X-ray diffraction analysis of the CRISPR-Cas RNA-silencing Cmr complex. Acta Crystallogr. F Struct. Biol. Commun. 71: 735–740. 26057804 10.1107/S2053230X15007104 PMC4461339

[bib37] Osawa T. Inanaga H. Sato C. Numata T., 2015 b Crystal structure of the CRISPR-Cas RNA silencing Cmr complex bound to a target analog. Mol. Cell 58: 418–430. 25921071 10.1016/j.molcel.2015.03.018

[bib38] Peng W. Feng M. Feng X. Liang Y. X. She Q., 2015 An archaeal CRISPR type III-B system exhibiting distinctive RNA targeting features and mediating dual RNA and DNA interference. Nucleic Acids Res. 43: 406–417. 25505143 10.1093/nar/gku1302 PMC4288192

[bib39] Pfaffl M. W., 2001 A new mathematical model for relative quantification in real-time RT-PCR. Nucleic Acids Res. 29: e45. 11328886 10.1093/nar/29.9.e45 PMC55695

[bib40] Qi L. S. Larson M. H. Gilbert L. A. Doudna J. A. Weissman J. S., 2013 Repurposing CRISPR as an RNA-guided platform for sequence-specific control of gene expression. Cell 152: 1173–1183. 23452860 10.1016/j.cell.2013.02.022 PMC3664290

[bib41] Rath D. Amlinger L. Hoekzema M. Devulapally P. R. Lundgren M., 2015 Efficient programmable gene silencing by Cascade. Nucleic Acids Res. 43: 237–246. 10.1093/nar/gku1257 PMC4288158 25435544

[bib42] Samai P. Pyenson N. Jiang W. Goldberg G. W. Hatoum-Aslan A., 2015 Co-transcriptional DNA and RNA cleavage during Type III CRISPR-Cas immunity. Cell 161: 1164–1174. 25959775 10.1016/j.cell.2015.04.027 PMC4594840

[bib43] Schleper C. Kubo K. Zillig W., 1992 The particle SSV1 from the extremely thermophilic archaeon *Sulfolobus* is a virus: demonstration of infectivity and of transfection with viral DNA. Proc. Natl. Acad. Sci. USA 89(16): 7645–7649. 1502176 10.1073/pnas.89.16.7645 PMC49767

[bib44] Shalem O. Sanjana N. E. Hartenian E. Shi X. Scott D. A., 2014 Genome-scale CRISPR-Cas9 knockout screening in human cells. Science 343: 84–87. 24336571 10.1126/science.1247005 PMC4089965

[bib45] Shmakov S. Abudayyeh O. O. Makarova K. S. Wolf Y. I. Gootenberg J. S., 2015 Discovery and functional characterization of diverse class 2 CRISPR-Cas systems. Mol. Cell 60: 385–397. 26593719 10.1016/j.molcel.2015.10.008 PMC4660269

[bib46] Staals R. H. Agari Y. Maki-Yonekura S. Zhu Y. Taylor D. W., 2013 Structure and activity of the RNA-targeting Type III-B CRISPR-Cas complex of *Thermus thermophilus*. Mol. Cell 52: 135–145. 24119403 10.1016/j.molcel.2013.09.013 PMC4006948

[bib47] Tamulaitis G. Kazlauskiene M. Manakova E. Venclovas C. Nwokeoji A. O., 2014 Programmable RNA Shredding by the Type III-A CRISPR-Cas System of *Streptococcus thermophilus*. Mol. Cell 56: 506–517. 25458845 10.1016/j.molcel.2014.09.027

[bib48] Taylor D. W. Zhu Y. Staals R. H. Kornfeld J. E. Shinkai A., 2015 Structural biology. Structures of the CRISPR-Cmr complex reveal mode of RNA target positioning. Science 348: 581–585. 25837515 10.1126/science.aaa4535 PMC4582657

[bib49] Worthington P. Blum P. Perez-Pomares F. Elthon T., 2003 Large-scale cultivation of acidophilic hyperthermophiles for recovery of secreted proteins. Appl. Environ. Microbiol. 69: 252–257. 12514002 10.1128/AEM.69.1.252-257.2003 PMC152466

[bib50] Wright A. V. Nunez J. K. Doudna J. A., 2016 Biology and applications of CRISPR systems: harnessing nature’s toolbox for genome engineering. Cell 164: 29–44. 26771484 10.1016/j.cell.2015.12.035

[bib51] Xiao Z. Storms R. Tsang A., 2006 A quantitative starch-iodine method for measuring alpha-amylase and glucoamylase activities. Anal. Biochem. 351: 146–148. 16500607 10.1016/j.ab.2006.01.036

[bib52] Zebec Z. Manica A. Zhang J. White M. F. Schleper C., 2014 CRISPR-mediated targeted mRNA degradation in the archaeon *Sulfolobus solfataricus*. Nucleic Acids Res. 42: 5280–5288. 24603867 10.1093/nar/gku161 PMC4005642

[bib53] Zetsche B. Gootenberg J. S. Abudayyeh O. O. Slaymaker I. M. Makarova K. S., 2015 Cpf1 is a single RNA-guided endonuclease of a class 2 CRISPR-Cas system. Cell 163: 759–771. 26422227 10.1016/j.cell.2015.09.038 PMC4638220

[bib54] Zhang J. Rouillon C. Kerou M. Reeks J. Brugger K., 2012 Structure and mechanism of the CMR complex for CRISPR-mediated antiviral immunity. Mol. Cell 45: 303–313. 22227115 10.1016/j.molcel.2011.12.013 PMC3381847

[bib55] Zhang J. Graham S. Tello A. Liu H. White M. F., 2016 Multiple nucleic acid cleavage modes in divergent type III CRISPR systems. Nucleic Acids Res 44: 1789–1799. 10.1093/nar/gkw020 PMC4770243 26801642

