# Supplementary material for: Efficient CRISPR-Mediated Post-Transcriptional Gene Silencing in a Hyperthermophilic Archaeon Using Multiplexed crRNA Expression
Source: G3 (Bethesda). 2016 Aug 8;6(10):3161–8. doi: 10.1534/g3.116.032482 (PMC5068938; doi:10.1534/g3.116.032482)
Supplement: Supplemental Material [file supp_6_10_3161__index.html]

Efficient CRISPR-Mediated Post-Transcriptional Gene Silencing in a Hyperthermophilic Archaeon Using Multiplexed crRNA Expression — Supplemental Material 

# Efficient CRISPR-Mediated Post-Transcriptional Gene Silencing in a Hyperthermophilic Archaeon Using Multiplexed crRNA Expression

## Supplemental Material for Zebec, *et al*, 2016

**Files in this Data Supplement:**

- File S1 - This file contains all Supplemental Material items as well as a supplemental reference list. (.pdf, 549 KB)
- Figure S1 - Schematic representation of multiplex miniCR-constructs analyzed in this study. (.pdf, 160 KB)
- Figure S2 - Schematic overview of the Modular OE-PCR used for construction of miniCR-AA123 as described in Materials and Methods. (.pdf, 292 KB)
- Figure S3 - Sequence of miniCR-AA12345 with length of each sequence part indicated. (.pdf, 155 KB)
- Figure S4 - Quantification of viral copies per chromosome of *S. solfataricus* miniCR-transformants. (.pdf, 118 KB)
- Table S1 - Information on PCR (polymerase chain reactions) used in this study. (.pdf, 120 KB)
- Table S2 - Sequences of primers used in this study. (.pdf, 17 KB)
