# Supplementary material for: Efficient CRISPR-Mediated Post-Transcriptional Gene Silencing in a Hyperthermophilic Archaeon Using Multiplexed crRNA Expression
Source: G3 (Bethesda). 2016 Aug 8;6(10):3161–8. doi: 10.1534/g3.116.032482 (PMC5068938; doi:10.1534/g3.116.032482)
Supplement: Supplemental Material [file supp_g3.116.032482_FileS1.pdf]

Efficient CRISPR-mediated posttranscriptional gene silencing in a hyperthermophilic archaeon using multiplexed crRNA expression

Supplementary information:

Figure S1, S2A, S2B, S3, S4

Table S1, S2

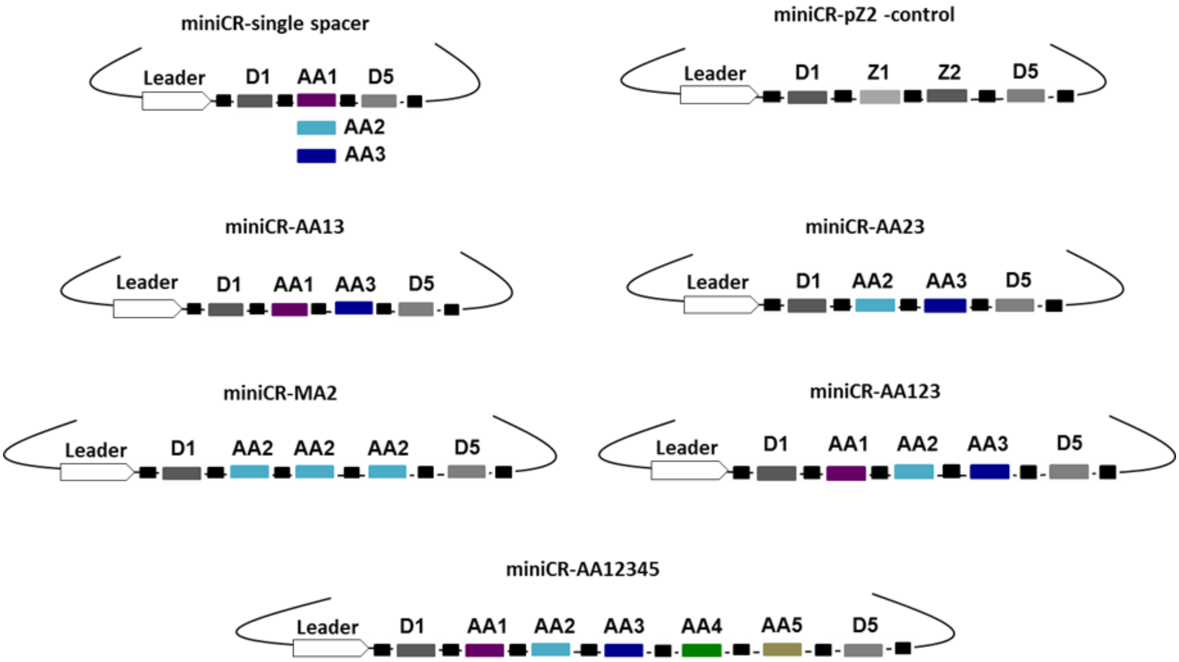

Figure S1. Schematic representation of multiplex miniCR-constructs analyzed in this study. All miniCR carry the leader of CRISPR locus D of *S. solfataricus* as promoter (white block arrow), locus D - specific repeats (black rectangles) and chromosomal spacers D1 and D5 (grey rectangles), which were maintained for cloning reasons. Colored rectangles (AA1-AA5) represent artificial spacers matching the host α-amylase mRNA. Single miniCR constructs carry either spacer AA1, AA2 or AA3. Different combinations of two spacers are carried with miniCR-AA13 and miniCR-AA23 and three and five in constructs miniCR-AA123 and miniCR-AA12345, respectively. Construct miniCR-MA2 carries three identical copies of spacer AA2. The control construct miniCR-pZ2-control harbors two spacers not matching the α-amylase.

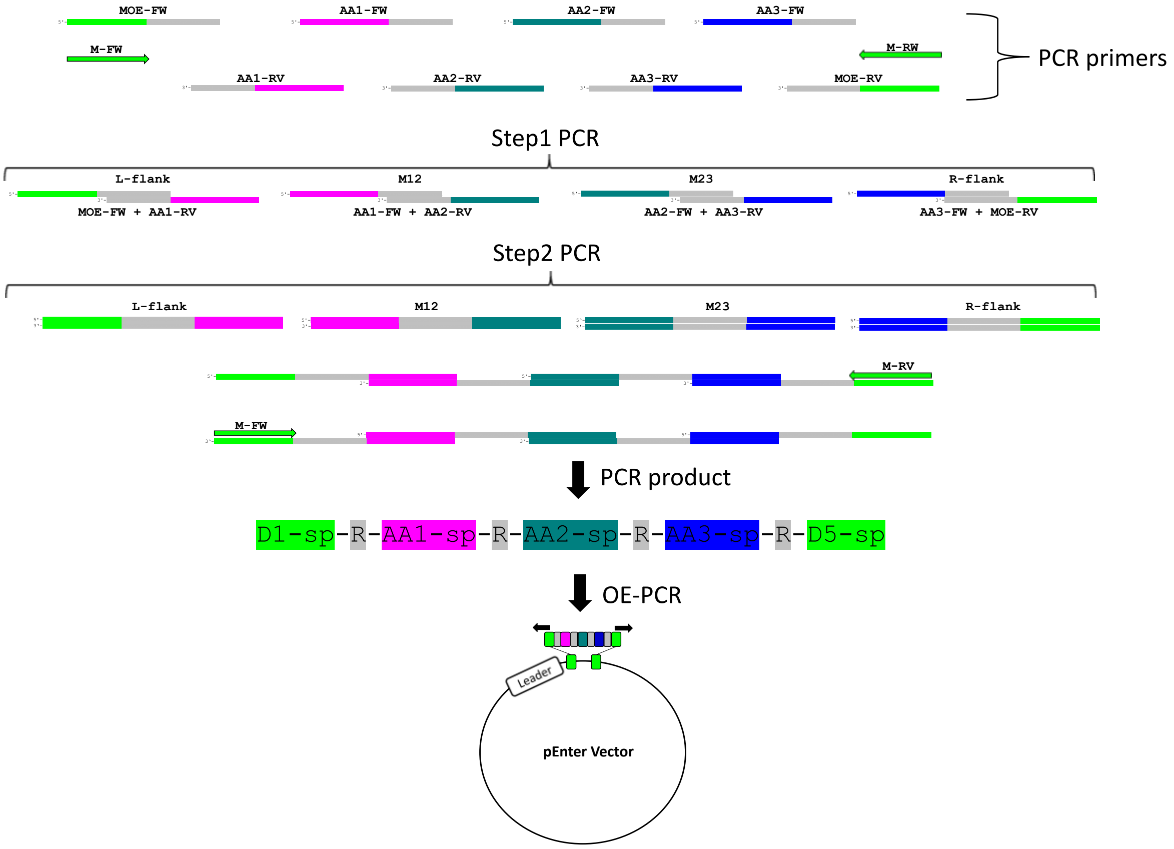

Figure S2A. Schematic overview of the Modular OE-PCR used for construction of miniCR-AA123 as described in Materials and Methods.

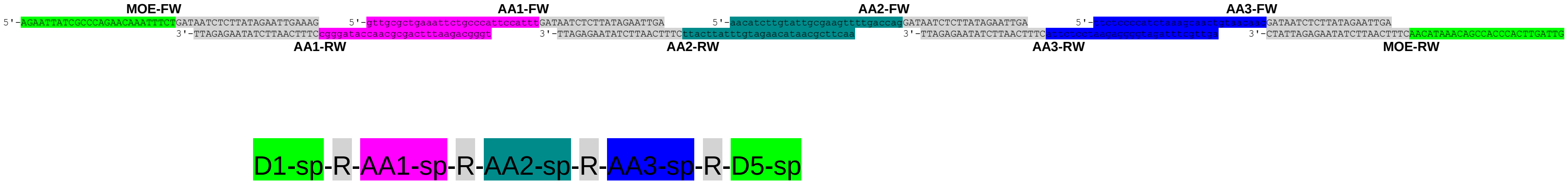

Figure S2B. Overlapping spacer sequences fused by overlap extension PCR (OE-PCR) to construct miniCR-AA123 according to the description in Materials and Methods.

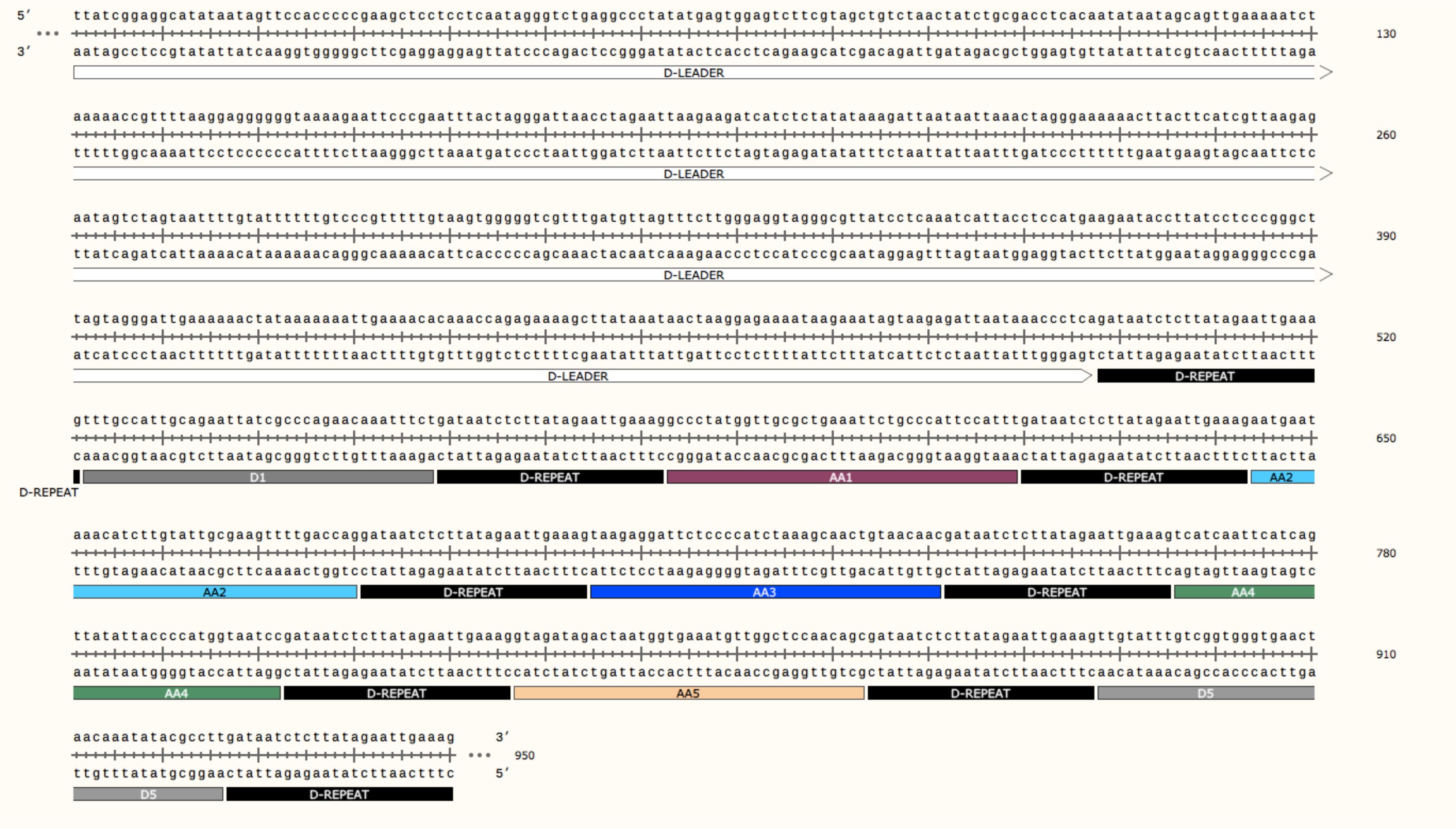

Figure S3. Sequence of miniCR-AA12345 with length of each sequence part indicated. D-leader (white) and D-repeats (black) are of the same sequence as in CRISPR locus D of *S. solfataricus* P2 and have a length of 497 bp and 24 bp, respectively. D1 and D5 spacers (grey) are also taken over from the host CRISPR-locus D and are of 37 bp and 39 bp, respectively. The artificial α-amylase targeting spacers (colored, AA1-AA5) are of 37 bp each. Map was designed using SnapGene Viewer software (from GSL Biotech; available at snapgene.com).

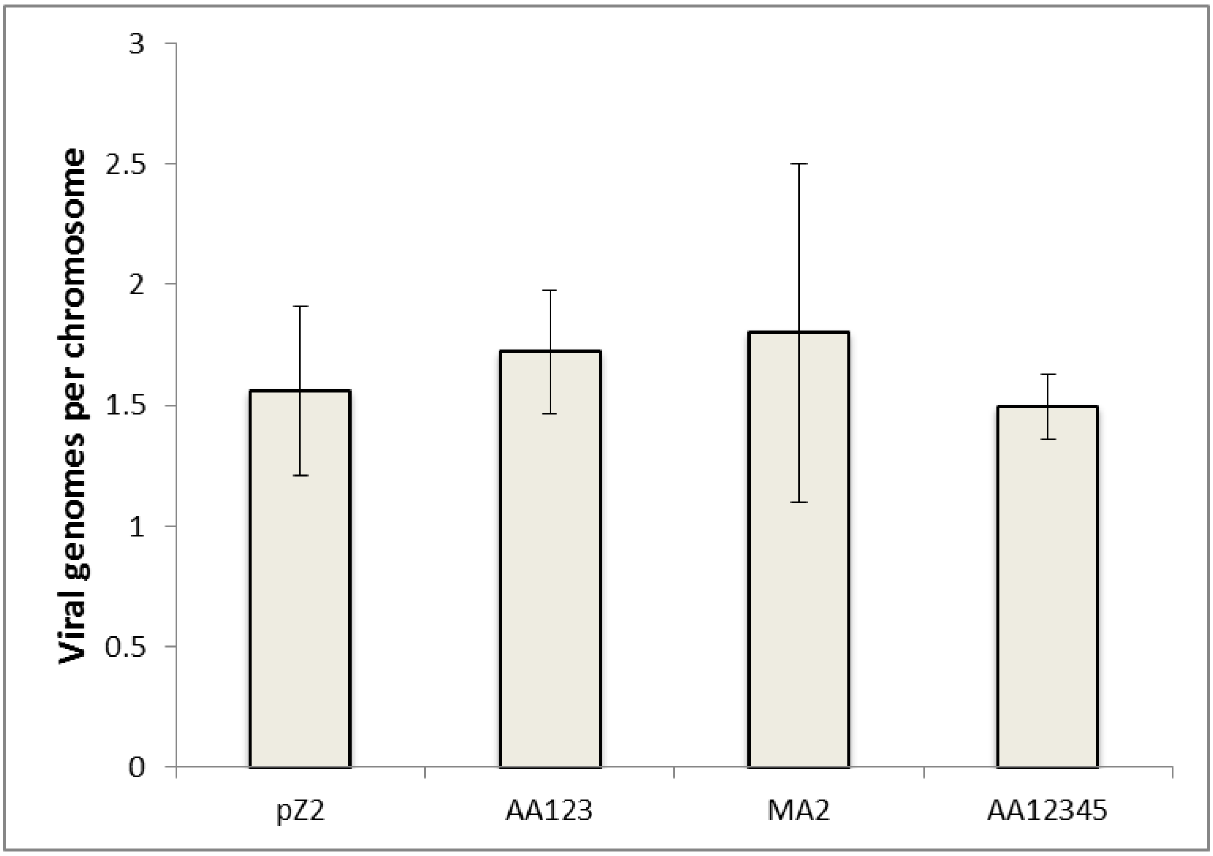

Figure S4. Quantification of viral copies per chromosome of *S. solfataricus* miniCR-transformants. Viral genomes as well as host chromosomes were measured via qPCR using primer pair Q- AA-Q2-no spacer (chromosome specific) and Q-A291 primers (virus DNA specific), respectively. Measurements of six biological replicates per construct are presented, whereas three of each were sampled at early - (t1) and three at late (t4) exponential growth, respectively. Error bars represent standard deviation (*SD*; $n \geq 3$). No significant difference was detected between samples and control pZ2 (two tailed t-test: $P \geq 0.38$ for all samples).

Table S1. Information on PCR (polymerase chain reactions) used in this study.

Primer name	Application	Product Size	Template	Construct
Q-AA2-sp_Fw	Q-PCR	612	α-amylase	/
Q-AA2-sp_Rv	Q-PCR	612	α-amylase	/
Q-AA2-no-sp_Fw	Q-PCR	290	α-amylase	/
Q-AA2-no-sp_Rv	Q-PCR	290	α-amylase	/
Q-Sso3194_Z_Fw	Q-PCR	280	Sso3194	/
Q-Sso3194_Z_Rv	Q-PCR	280	Sso3194	/
CR6-Fw	PCR	986 max.	CR6	/
CR6-Rv	PCR	986 max.	CR6	/
M_Fw	OE	/	miniCR	All miniCR
M_Rv	OE	/	miniCR	All miniCR
MOE_Fw	OE	/	miniCR	All miniCR
MOE_Rv	OE	/	miniCR	All miniCR
AA5_Fw	OE	/	miniCR	miniCR
AA5_Rv	OE	/	miniCR	miniCR
AA5_Fw	OE	/	miniCR	miniCR
AA5_Rv	OE	/	miniCR	miniCR
AA5_Fw	OE	/	miniCR	miniCR
AA5_Rv	OE	/	miniCR	miniCR
AA4_Fw	OE	/	miniCR	miniCR
AA4_Rv	OE	/	miniCR	miniCR
AA5_Fw	OE	/	miniCR	miniCR
AA5_Rv	OE	/	miniCR-AA2	miniCR
MA2-over_Fw	Inverse PCR	3090	miniCR-AA2	miniCR-MA2
MA2-over_Rv	Inverse PCR	3090	miniCR-AA2	miniCR-MA2
MA2-lin_Fw	Inverse PCR	3000	miniCR-AA2	miniCR-MA2
MA2-lin_Rv	Inverse PCR	3000	miniCR-AA2	miniCR-MA2

Table S2. Sequences of primers used in this study.

Primer name	Sequence 5'-3'
Q-AA2-sp_Fw	GGGCTAACTACTGGACCCCA
Q-AA2-sp_Rv	GTGTAACCATACCCAAGGTTGCT
Q-AA2-no-sp_Fw	TTTCGATTCAGATCGCTGGCAA
Q-AA2-no-sp_Rv	GTGTAACCATACCCAAGGTTGCT
Q-Sso3194_Z_Fw	ATCAGTGGAGACGAGTGGCAAGA
Q-Sso3194_Z_Rv	ATTGCAGCCTTAACCTCGCCTTCT
CR6-Fw	TTATCGGAGGCATATAATAGTTCCA
CR6-Rv	AATCCAATGAGCCGGGACAAGTTTCACAA
M_Fw	TGCAGAATTATCGCCCAGAACAA
M_Rv	GTTAGTTCACCCACCGACAAATACA
MOE_Fw	AGAATTATCGCCCAGAACAAATTTCTGATAATCTCTTATAGAATTGAAAG
MOE_Rv	GTTAGTTCACCCACCGACAAATACAACTTTCAATTCTATAAGAGATTATC
AA5_Fw	gttgcgctgaaattctgcccattccatttGATAATCTCTTATAGAATTGA
AA5_Rv	tgggcagaatttcagcgcaaccatagggcCTTTCAATTCTATAAGAGATT
AA5_Fw	aacatcttgtattgcgaagttttgaccagGATAATCTCTTATAGAATTGA
AA5_Rv	aacttcgcaatacaagatgtttattcattCTTTCAATTCTATAAGAGATT
AA5_Fw	ttctccccatctaaagcaactgtaacaacGATAATCTCTTATAGAATTGA
AA5_Rv	agttgctttagatggggagaatcctcttaCTTTCAATTCTATAAGAGATT
AA4_Fw	tcatcagttatattaccccatggtaatccGATAATCTCTTATAGAATTGA
AA4_Rv	atggggtaatataactgatgaattgatgaCTTTCAATTCTATAAGAGATT
AA5_Fw	actaatggtgaaatgttggctccaacagcGATAATCTCTTATAGAATTGA
AA5_Rv	AGCCAACATTTCACCATTAGTCTATCTACCTTTCAATTCTATAAGAGATT
MA2-over_Fw	TACAAGATGTTTATTCATTCTTTCAATTCTATAAGAGATTATCCTGGTCAAAACTTCGCAATACAAGATGTTTATTCATTCTTTCA
MA2-over_Rv	ATTGCGAAGTTTTGACCAGGATAATCTCTTATAGAATTGAAAGAATGAATAAACATCTTGTATTGCGAAGTTTTGACCAG
MA2-lin_Fw	ACAAGATGTTTATTCATTCTTTCA
MA2-lin_Rv	ATTGCGAAGTTTTGACCAG

Supplementary Material Reference

1. Haseltine C, Rolfsmeier M, Blum P. The glucose effect and regulation of alpha-amylase synthesis in the hyperthermophilic archaeon Sulfolobus solfataricus. Journal of bacteriology 1996; 178:945-50.
