# Supplementary material for: Efficient CRISPR-Mediated Post-Transcriptional Gene Silencing in a Hyperthermophilic Archaeon Using Multiplexed crRNA Expression
Source: G3 (Bethesda). 2016 Aug 8;6(10):3161–8. doi: 10.1534/g3.116.032482 (PMC5068938; doi:10.1534/g3.116.032482)
Supplement: Supplemental Material [file supp_g3.116.032482_FigureS1.pdf]

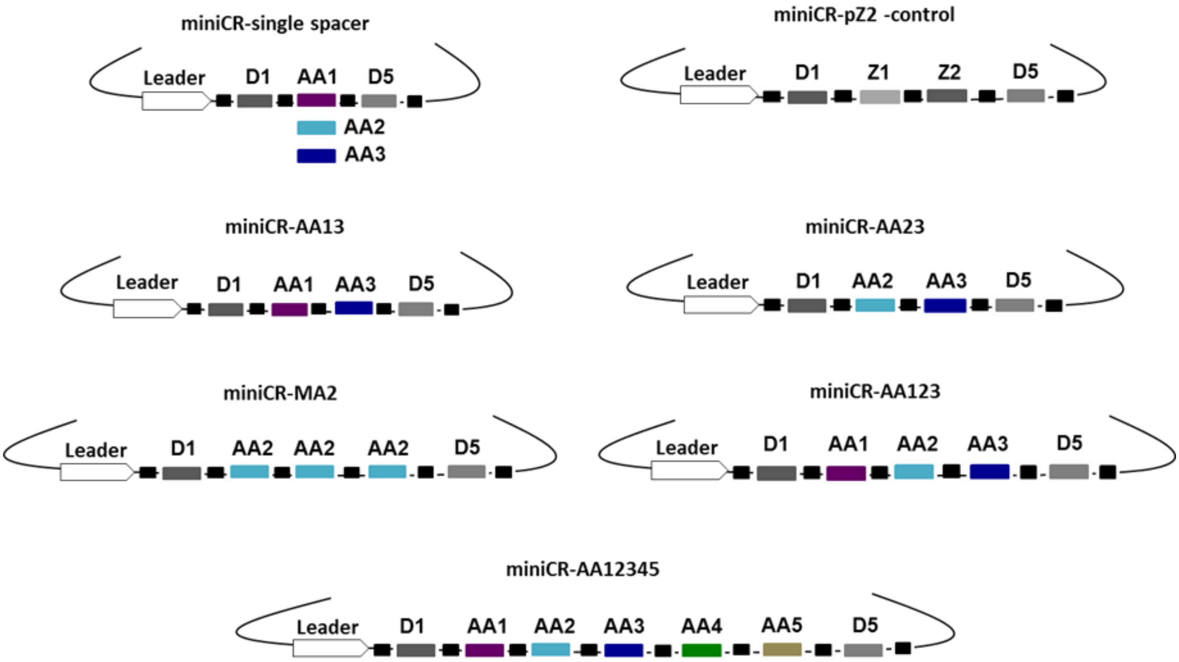


**Figure S1.** Schematic representation of multiplex miniCR-constructs analyzed in this study. All miniCR carry the leader of CRISPR locus D of *S. solfataricus* as promoter (white block arrow), locus D - specific repeats (black rectangles) and chromosomal spacers D1 and D5 (grey rectangles), which were maintained for cloning reasons. Colored rectangles (AA1-AA5) represent artificial spacers matching the host α-amylase mRNA. Single miniCR constructs carry either spacer AA1, AA2 or AA3. Different combinations of two spacers are carried with miniCR-AA13 and miniCR-AA23 and three and five in constructs miniCR-AA123 and miniCR-AA12345, respectively. Construct miniCR-MA2 carries three identical copies of spacer AA2. The control construct miniCR-pZ2-control harbors two spacers not matching the α-amylase.
