# Supplementary material for: Efficient CRISPR-Mediated Post-Transcriptional Gene Silencing in a Hyperthermophilic Archaeon Using Multiplexed crRNA Expression
Source: G3 (Bethesda). 2016 Aug 8;6(10):3161–8. doi: 10.1534/g3.116.032482 (PMC5068938; doi:10.1534/g3.116.032482)
Supplement: Supplemental Material [file supp_g3.116.032482_FigureS2.pdf]

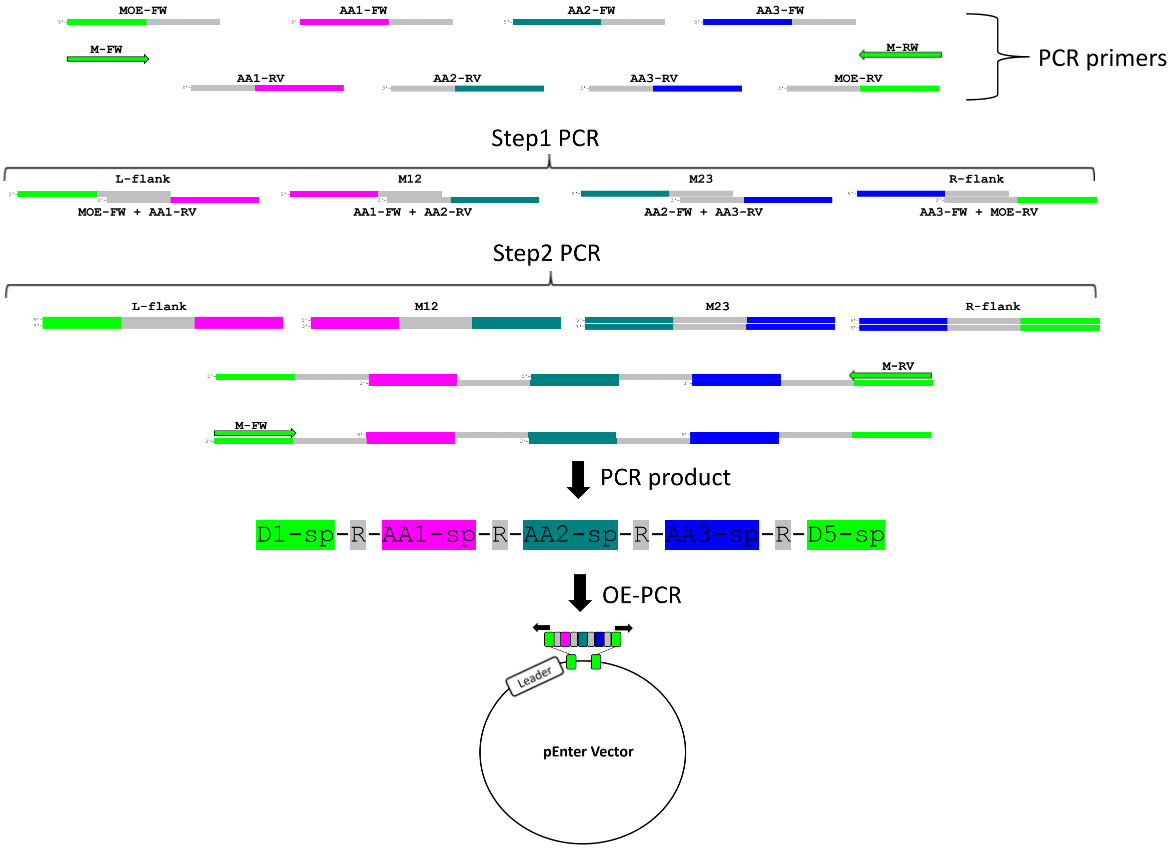

Figure S2A. Schematic overview of the Modular OE-PCR used for construction of miniCR-AA123 as described in Materials and Methods.

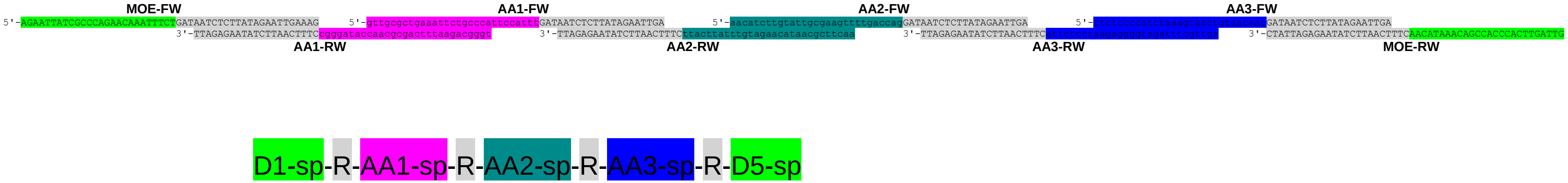

Figure S2B. Overlapping spacer sequences fused by overlap extension PCR (OE-PCR) to construct miniCR-AA123 according to the description in Materials and Methods.
