# Supplementary material for: Efficient CRISPR-Mediated Post-Transcriptional Gene Silencing in a Hyperthermophilic Archaeon Using Multiplexed crRNA Expression
Source: G3 (Bethesda). 2016 Aug 8;6(10):3161–8. doi: 10.1534/g3.116.032482 (PMC5068938; doi:10.1534/g3.116.032482)
Supplement: Supplemental Material [file supp_g3.116.032482_FigureS3.pdf]

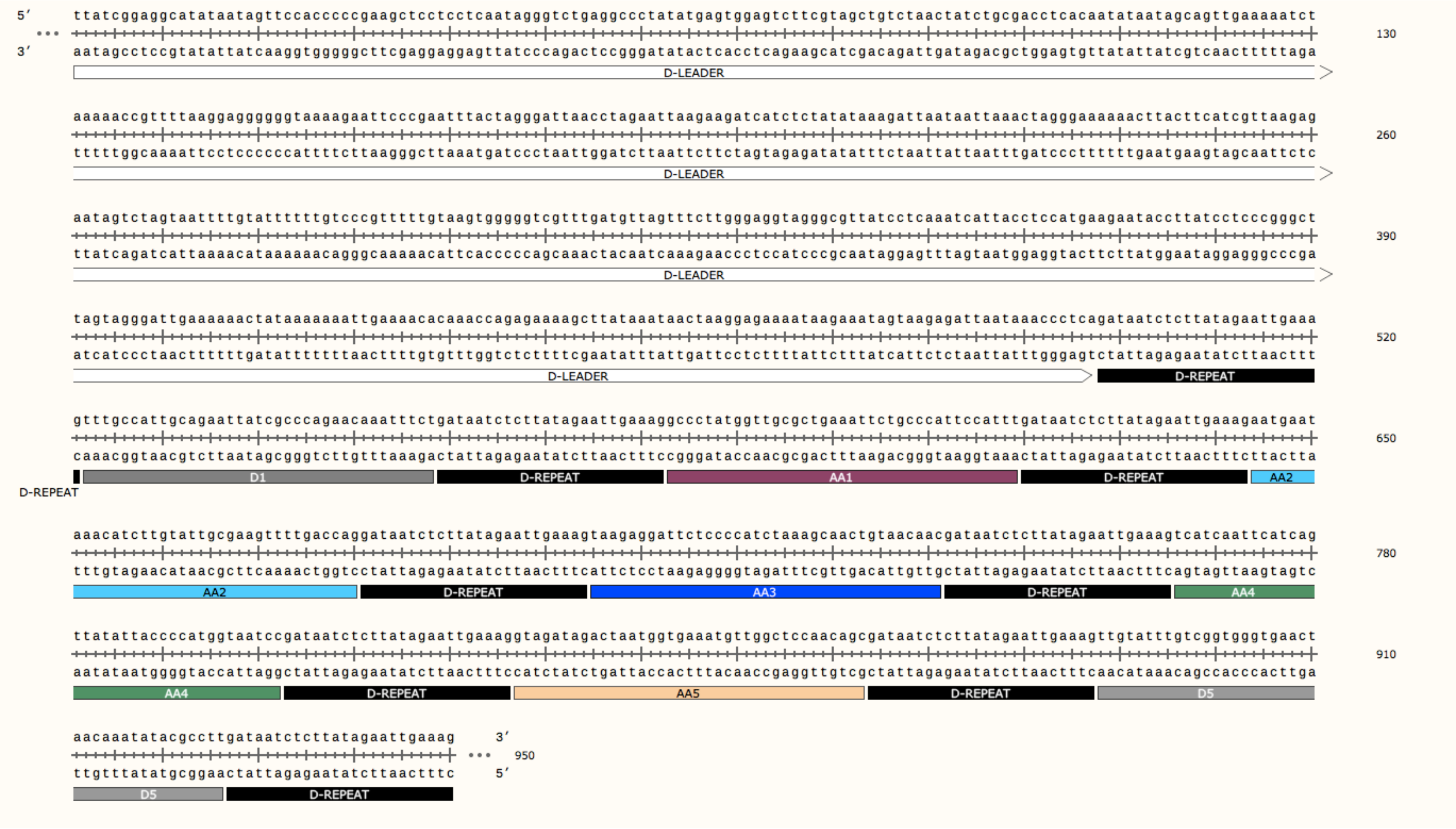

Figure S3. Sequence of miniCR-AA12345 with length of each sequence part indicated. D-leader (white) and D-repeats (black) are of the same sequence as in CRISPR locus D of *S. solfataricus* P2 and have a length of 497 bp and 24 bp, respectively. D1 and D5 spacers (grey) are also taken over from the host CRISPR-locus D and are of 37 bp and 39 bp, respectively. The artificial α-amylase targeting spacers (colored, AA1-AA5) are of 37 bp each. Map was designed using SnapGene Viewer software (from GSL Biotech; available at snapgene.com).
