# Supplementary material for: Efficient CRISPR-Mediated Post-Transcriptional Gene Silencing in a Hyperthermophilic Archaeon Using Multiplexed crRNA Expression
Source: G3 (Bethesda). 2016 Aug 8;6(10):3161–8. doi: 10.1534/g3.116.032482 (PMC5068938; doi:10.1534/g3.116.032482)
Supplement: Supplemental Material [file supp_g3.116.032482_FigureS4.pdf]

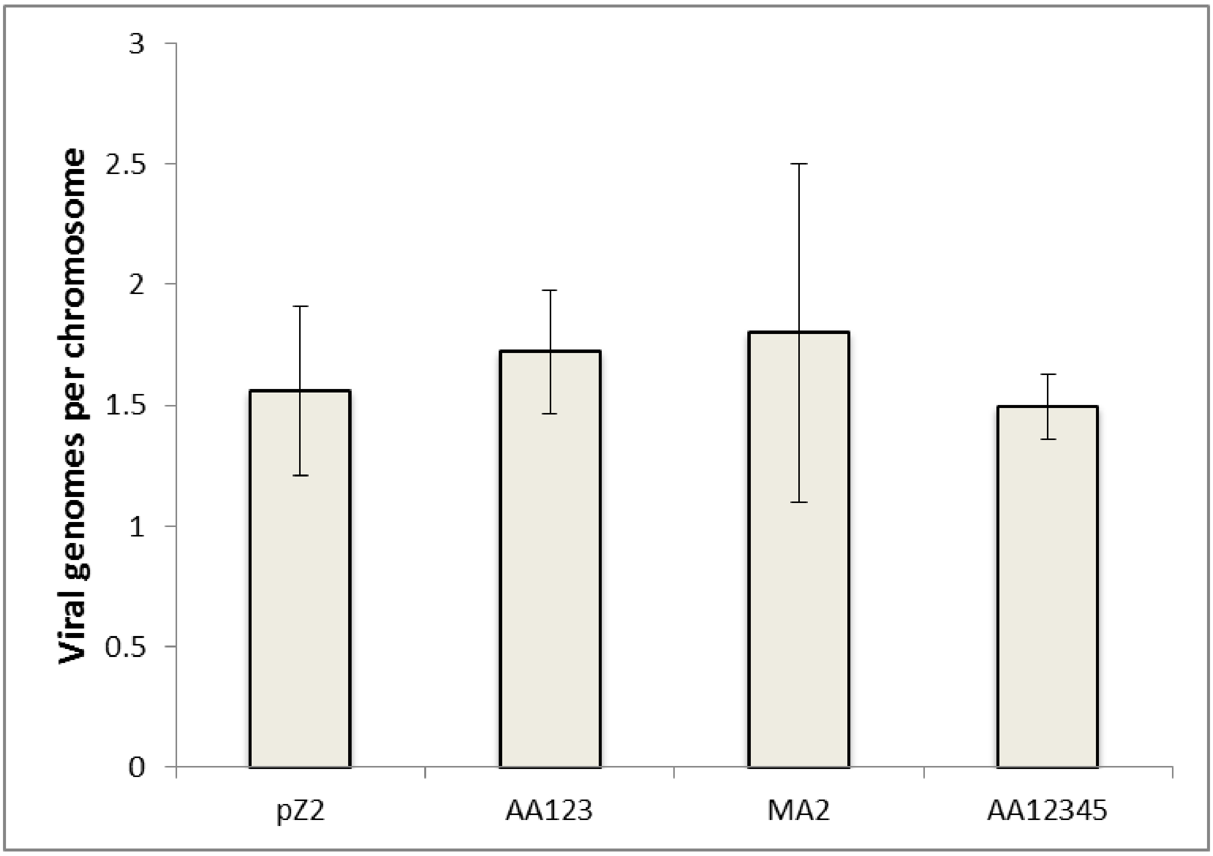

Figure S4. Quantification of viral copies per chromosome of *S. solfataricus* miniCR-transformants. Viral genomes as well as host chromosomes were measured via qPCR using primer pair Q- AA-Q2-no spacer (chromosome specific) and Q-A291 primers (virus DNA specific), respectively. Measurements of six biological replicates per construct are presented, whereas three of each were sampled at early - (t1) and three at late (t4) exponential growth, respectively. Error bars represent standard deviation (*SD*; $n \geq 3$). No significant difference was detected between samples and control pZ2 (two tailed t-test: $P \geq 0.38$ for all samples).
