# Supplementary material for: Efficient CRISPR-Mediated Post-Transcriptional Gene Silencing in a Hyperthermophilic Archaeon Using Multiplexed crRNA Expression
Source: G3 (Bethesda). 2016 Aug 8;6(10):3161–8. doi: 10.1534/g3.116.032482 (PMC5068938; doi:10.1534/g3.116.032482)
Supplement: Supplemental Material [file supp_g3.116.032482_TableS1.pdf]

Table S1. Information on PCR (polymerase chain reactions) used in this study.

Primer name	Application	Product Size	Template	Construct
Q-AA2-sp_Fw	Q-PCR	612	α-amylase	/
Q-AA2-sp_Rv	Q-PCR	612	α-amylase	/
Q-AA2-no-sp_Fw	Q-PCR	290	α-amylase	/
Q-AA2-no-sp_Rv	Q-PCR	290	α-amylase	/
Q-Sso3194_Z_Fw	Q-PCR	280	Sso3194	/
Q-Sso3194_Z_Rv	Q-PCR	280	Sso3194	/
CR6-Fw	PCR	986 max.	CR6	/
CR6-Rv	PCR	986 max.	CR6	/
M_Fw	OE	/	miniCR	All miniCR
M_Rv	OE	/	miniCR	All miniCR
MOE_Fw	OE	/	miniCR	All miniCR
MOE_Rv	OE	/	miniCR	All miniCR
AA5_Fw	OE	/	miniCR	miniCR
AA5_Rv	OE	/	miniCR	miniCR
AA5_Fw	OE	/	miniCR	miniCR
AA5_Rv	OE	/	miniCR	miniCR
AA5_Fw	OE	/	miniCR	miniCR
AA5_Rv	OE	/	miniCR	miniCR
AA4_Fw	OE	/	miniCR	miniCR
AA4_Rv	OE	/	miniCR	miniCR
AA5_Fw	OE	/	miniCR	miniCR
AA5_Rv	OE	/	miniCR-AA2	miniCR
MA2-over_Fw	Inverse PCR	3090	miniCR-AA2	miniCR-MA2
MA2-over_Rv	Inverse PCR	3090	miniCR-AA2	miniCR-MA2
MA2-lin_Fw	Inverse PCR	3000	miniCR-AA2	miniCR-MA2
MA2-lin_Rv	Inverse PCR	3000	miniCR-AA2	miniCR-MA2
