# Supplementary material for: Efficient CRISPR-Mediated Post-Transcriptional Gene Silencing in a Hyperthermophilic Archaeon Using Multiplexed crRNA Expression
Source: G3 (Bethesda). 2016 Aug 8;6(10):3161–8. doi: 10.1534/g3.116.032482 (PMC5068938; doi:10.1534/g3.116.032482)
Supplement: Supplemental Material [file supp_g3.116.032482_TableS2.pdf]

Table S2. Sequences of primers used in this study.

Primer name	Sequence 5'-3'
Q-AA2-sp_Fw	GGGCTAACTACTGGACCCCA
Q-AA2-sp_Rv	GTGTAACCATACCCAAGGTTGCT
Q-AA2-no-sp_Fw	TTTCGATTCAGATCGCTGGCAA
Q-AA2-no-sp_Rv	GTGTAACCATACCCAAGGTTGCT
Q-Sso3194_Z_Fw	ATCAGTGGAGACGAGTGGCAAGA
Q-Sso3194_Z_Rv	ATTGCAGCCTTAACCTCGCCTTCT
CR6-Fw	TTATCGGAGGCATATAATAGTTCCA
CR6-Rv	AATCCAATGAGCCGGGACAAGTTTCACAA
M_Fw	TGCAGAATTATCGCCCAGAACAA
M_Rv	GTTAGTTCACCCACCGACAAATACA
MOE_Fw	AGAATTATCGCCCAGAACAAATTTCTGATAATCTCTTATAGAATTGAAAG
MOE_Rv	GTTAGTTCACCCACCGACAAATACAACTTTCAATTCTATAAGAGATTATC
AA5_Fw	gttgcgctgaaattctgcccattccatttGATAATCTCTTATAGAATTGA
AA5_Rv	tgggcagaatttcagcgcaaccatagggcCTTTCAATTCTATAAGAGATT
AA5_Fw	aacatcttgtattgcgaagttttgaccagGATAATCTCTTATAGAATTGA
AA5_Rv	aacttcgcaatacaagatgtttattcattCTTTCAATTCTATAAGAGATT
AA5_Fw	ttctccccatctaaagcaactgtaacaacGATAATCTCTTATAGAATTGA
AA5_Rv	agttgctttagatggggagaatcctcttaCTTTCAATTCTATAAGAGATT
AA4_Fw	tcatcagttatattaccccatggtaatccGATAATCTCTTATAGAATTGA
AA4_Rv	atggggtaatataactgatgaattgatgaCTTTCAATTCTATAAGAGATT
AA5_Fw	actaatggtgaaatgttggctccaacagcGATAATCTCTTATAGAATTGA
AA5_Rv	AGCCAACATTTCACCATTAGTCTATCTACCTTTCAATTCTATAAGAGATT
MA2-over_Fw	TACAAGATGTTTATTCATTCTTTCAATTCTATAAGAGATTATCCTGGTCAAAACTTCGCAATACAAGATGTTTATTCATTCTTTCA
MA2-over_Rv	ATTGCGAAGTTTTGACCAGGATAATCTCTTATAGAATTGAAAGAATGAATAAACATCTTGTATTGCGAAGTTTTGACCAG
MA2-lin_Fw	ACAAGATGTTTATTCATTCTTTCA
MA2-lin_Rv	ATTGCGAAGTTTTGACCAG
